# AmelHap: Leveraging drone whole-genome sequence data to create a honey bee HapMap

**DOI:** 10.1038/s41597-023-02097-z

**Published:** 2023-04-10

**Authors:** M. Parejo, A. Talenti, M. Richardson, A. Vignal, M. Barnett, D. Wragg

**Affiliations:** 1 Applied Genomics and Bioinformatics, University of the Basque Country (UPV/EHU), Leioa, Spain; 2 The Roslin Institute, University of Edinburgh, Easter Bush Campus, Midlothian, UK; 3 University of Edinburgh, King’s Buildings Campus, Edinburgh, UK; 4 Beebytes Analytics CIC, Roslin Innovation Centre, Easter Bush Campus, Midlothian, UK; 5 GenPhySE, Université de Toulouse, INRAE, INPT, INP-ENVT, 31326 Castanet Tolosan, France

**Keywords:** Haplotypes, Genetic variation

## Abstract

Honey bee, *Apis mellifera*, drones are typically haploid, developing from an unfertilized egg, inheriting only their queen’s alleles and none from the many drones she mated with. Thus the ordered combination or ‘phase’ of alleles is known, making drones a valuable haplotype resource. We collated whole-genome sequence data for 1,407 drones, including 45 newly sequenced Scottish drones, collectively representing 19 countries, 8 subspecies and various hybrids. Following alignment to Amel_HAv3.1, variant calling and quality filtering, we retained 17.4 M high quality variants across 1,328 samples with a genotyping rate of 98.7%. We demonstrate the utility of this haplotype resource, AmelHap, for genotype imputation, returning >95% concordance when up to 61% of data is missing in haploids and up to 12% of data is missing in diploids. AmelHap will serve as a useful resource for the community for imputation from low-depth sequencing or SNP chip data, accurate phasing of diploids for association studies, and as a comprehensive reference panel for population genetic and evolutionary analyses.

## Background & Summary

The honey bee, *Apis mellifera*, is the most important managed pollinator for crop and wild flora worldwide^1,2^. Since the publication of the honey bee genome in 2006 it has received much attention by the scientific community, resulting in an increasing number of papers on honey bee genomics^3^. In fact, numerous whole-genome sequencing studies have been published since, studying honey bee population structure and diversity^4–8^, evolutionary history^9,10^, and investigating signatures of selection^11–13^ not only in extant populations, but also museum specimens^14^. Mining whole-genome sequence data sets has also been used to identify ancestry informative markers, for instance, and to quantify admixture^4,15^ and identify subspecies^6^ for breeding or conservation purposes. Besides, whole-genome sequencing has also been used for quantitative-trait loci (QTL) mapping of economically important traits^16–18^, as well as genome-environment associations to identify local adaptations^19–21^.

Despite the decreasing costs of genome-sequencing, large-scale population genomics studies are still not feasible for all labs, and thus, studies are often limited by sample size^7^. Large sample sizes are especially important to increase power for genotype-phenotype associations^22,23^. Moreover, accuracy of QTL mapping and genome-wide associations studies greatly benefit from a haplotype-resolved dataset^24,25^, in particular, to identify structural variation^26,27^. For honey bees, structural variation has been shown to be characteristic in the differentiation of subspecies and local adaptation^8,28^, but accurate phasing empowered by a large haplotype reference is necessary - in particular due to the honey bee’s high recombination rate^29^. More recently in other species, pangenomes are emerging as a powerful tool to characterise structural variation opening up previously inaccessible genomic regions^30,31^. The development of a pangenome for the honey bee would therefore benefit from a large and diverse dataset of reference haplotypes.

For these reasons, we have collated and curated whole-genome sequence data from 1,407 drones representing a diverse range of subspecies, habitats and geographic origins, into an accessible genomic resource: AmelHap. The key aims of AmelHap are to support genotype imputation, haplotype phasing and act as a comprehensive reference panel, similar to the human International HapMap Project^32^. Significant advantages of *A. mellifera* for such a project include its small genome size (225.25 Mb; Amel_HAv3.1) and the availability of haploid drones whose alleles are naturally phased. The resource will be useful for a variety of applications, including for example: imputation of low-depth sequence or SNP array data to enable cost-efficient large-scale studies; accurate phasing of diploid genomes to facilitate haplotype-based analyses such as XP-EHH;^33^ recovery of host genomic data from suboptimal samples (such as bee hive products^34^, metagenomic^35^, historic or ancient DNA^14^); identification of ancestry informative markers or tag-SNPs; validation of reduced SNP panels; and to serve as a comprehensive reference panel to support studies on population and evolutionary genetics.

Herein, we *i)* describe the details of the AmelHap dataset including sample origins and sample strategies, *ii)* outline step-by-step our sequence processing pipeline, and *iii)* validate AmelHap on its performance for genotype imputation in a haploid and diploid dataset. We have created a Zenodo community (https://zenodo.org/communities/amelhap) for the ongoing development of AmelHap, where we have released the metadata, raw and filtered variant data from this study.

## Methods

### Sample origins

We processed publicly available Illumina sequencing data for 1,362 honey bee drones representing 19 countries, 4 distinct evolutionary lineages and 8 subspecies: *A. m. capensis*, *A. m. scutellata* and *A. m. unicolor* from the African (A) lineage; *A. m. carnica* and *A. m. ligustica* from the central and southern European (C) lineage; *A. m. iberiensis* and *A. m. mellifera* from the northern and western European (M) lineage; and *A. m. caucasia* from the Eastern European lineage (O). The drones comprised 1,156 samples from Europe^4,5,11,36^, 73 from South Africa and the South West Indian Ocean^5,36^, 125 from North America^37^, 13 from Asia, and 40 from Oceania^8^. In addition to representatives of the 8 subspecies listed, the dataset comprises various hybrids including Buckfast and experimental populations from Canada and Gotland. An overview of the sample origins can be found in Supplementary Table 1, and further details on each dataset are provided below.

PRJNA311274^38^: This Bioproject includes a large number of drones sequenced for the French research project SeqApiPop - an initiative to characterise the genetic diversity of French honey bees that not only included populations from France but also reference samples from several other countries. To date, 3 studies have been published within the scope of SeqApiPop. An initial pilot study investigated selection signatures in commercial honey bee populations^11^. This included sequence data for 30 unrelated drones selected for royal jelly and another 32 for honey production, as well as 30 *A. m. mellifera* drones from the island of Ouessant, 12 *A. m. carnica* from Germany and 18 *A. m. carnica* from Slovenia. A second study, explored admixture between European and African honey bees in the South West Indian Ocean islands^5^. Sequence data sourced from this study included 6 unrelated drones from Reunion, 2 from Rodrigues, 6 from Madagascar, and 2 from Mauritius. Drones from Madagascar and Mauritius are reported to be typical examples of the endemic *A. m. unicolor*, while those on Rodrigues and Reunion are hybrids. In addition, data was generated for 10 *A. m. ligustica* drones from Italy, and 10 *A. m. caucasia* drones from a breeder that had imported colonies to France from Georgia. Finally, the most recent study from the SeqApiPop to characterize French bee populations provides sequences data for a further 681 drones from independent colonies across Europe including France, Spain, Germany, Switzerland, Italy, the UK, Slovenia, Poland, Denmark, China, and New Caledonia^8^. These drones include *A. m. mellifera* from conservatories, *A. m. carnica* from breeders and breeder organizations, *A. m. caucasia* and Buckfast from breeders in France, *A. m. iberiensis* from beekeepers in Spain, *A. m. ligustica* from breeders in Italy, as well as a large number of drones of hybrid genetic ancestry. These samples also include a large number from a *Varroa-*resistance study^39^ in Vaucluse (France) labelled the MOSAR experiment.

PRJNA596071^40^: Sequence data of 61 unrelated drones obtained from this study were sampled across Austria, Germany, Slovenia, Switzerland and Norway^16^. These include representatives of *A. m. carnica* and *A. m. mellifera* from international breeding and conservation programmes. The drones were sequenced in the frame of the development of a SNP array for genomic selection.

PRJNA363032^41^: Sequence data for 125 drones from Canada was sourced from a study of hygienic behaviour^37^. Drone larvae were sampled randomly from selected and control populations, averaging 3.1 drones per colony, totaling 41 colonies. Canadian honey bees are highly admixed, but derive predominantly from the C and M lineages^42^. Drones were sampled from the third generation of two artificially selected populations based on increased hygienic behaviour, as well as from an unselected baseline population.

PRJNA516678^43^: Sequence data for 158 drones from 8 colonies each on the islands of Gotland (Sweden) and Åland (Finland), in addition to 57 drones from 6 colonies in South Africa (*A. m. capensis*, n = 28; *A. m. scutellata*, n = 29), was sourced from a study of recombination^36^. Between 8 and 10 drones were sampled per colony across 22 colonies. The samples from Gotland originate from a natural selection experiment between 1997–1999, in which 150 colonies were introduced to the island, artificially infested with the mite *Varroa destructor*, and left unmanaged in a “live and let die” experiment referred to as the “Bond Project”^44,45^. The bulk of colonies introduced from Sweden were described as Buckfast, a hybrid strain mainly derived from the C lineage, in addition to 10 queens each sourced from Swedish *A. m. carnica* and *A. m. mellifera* breeders^44^. The Åland bees were described as typical managed honey bees, containing a mixture of European ancestry^36^.

PRJEB16533^46^: Sequence data of 119 unrelated drones, each sampled from a different colony, was retrieved from a study on conservation genomics in Switzerland^4^. Of these, 39 *A. m. mellifera* drones were sampled from Swiss conservation areas, while *A. m. carnica* drones (n = 33) and Buckfast drones (n = 14) were obtained from breeders and breeder associations of the respective strains.

PRJNA578233^47^: This project accession, which has no associated publication, comprises three *A. m. mellifera* drones collected from the South Urals (Bashkortostan, Russia), where a large population of pure dark honey bees are found^48^.

### Drone sampling and sequencing

In addition to publicly available data, we collected 5 drone pupae from each of 9 colonies from the Lothians in Scotland, totaling 45 samples of undefined ancestry (Bioproject: PRJEB39369^49^). DNA was extracted from the thorax of individual drones with Zymo Quick-DNA MiniPrep Kits, and quantified by Qubit. DNA samples were sequenced by Novogene (UK) on the Illumina^TM^ NovaSeq platform, following library preparation for 150 bp paired end reads, generating ~4 Gb of raw data per sample.

### Sequencing read alignment and variant calling

**Fig. 1:**
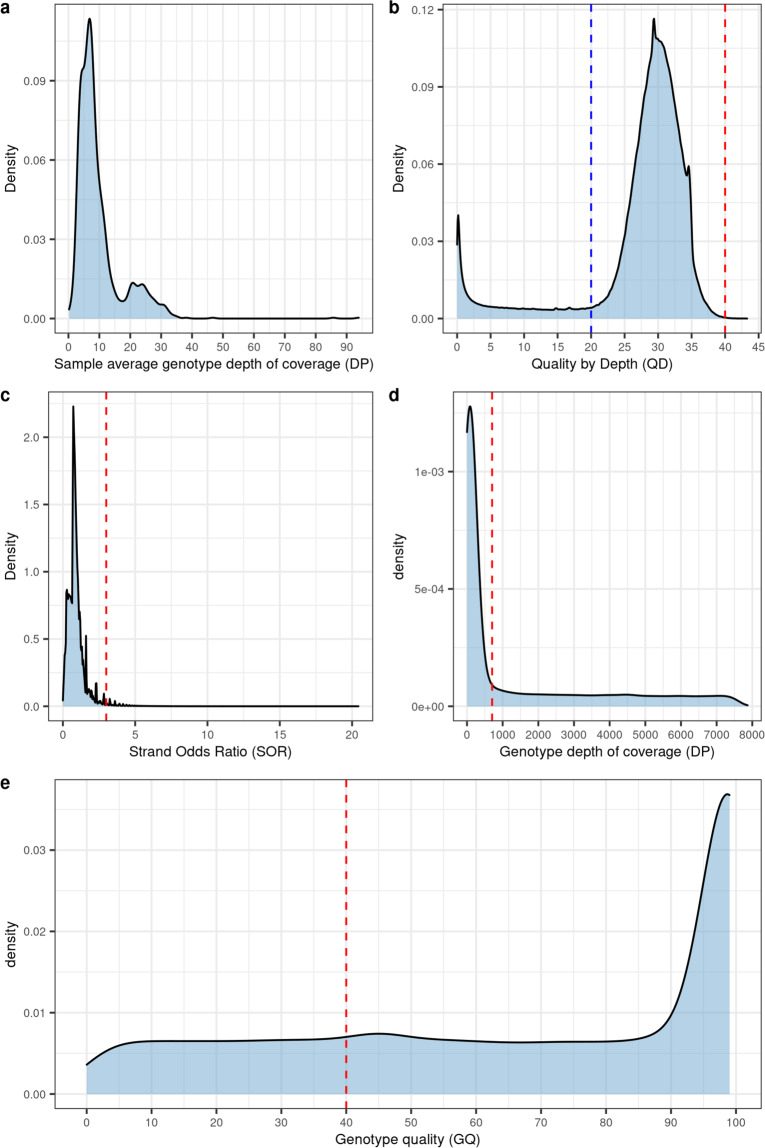
Variants and genotypes were subject to a range of filters based on the distributions of several key metrics. (**a**) Sample-level average genotype depth of coverage (DP), indicating a mean DP of 7.4 ± 7.5. (**b**) Quality by depth (QD) density plot. In downstream filtering we retain variants with 20 ≤ QD ≤ 40, denoted by dashed blue and red lines, with the aim to exclude variants potentially harbouring a heterozygous signature such as those in sequence duplications. (**c**) Strands odds ratio (SOR) density plot with dashed red line indicating threshold subsequently used to filter out variants with statistical strand bias (SOR > 3). After filtering to retain variant records with 20 ≤ QD ≤ 40, MQ ≥ 50, and SOR ≤ 3, we evaluated sample-level genotype depth of coverage (DP) and genotype quality (GQ). (**d**) Density plot of genotype DP, red line indicates threshold above which genotypes were subsequently set to missing (DP > 704). (**e**) Density plot of GQ, red line indicates threshold below which genotypes were subsequently set to missing (GQ < 40).

Sequencing reads were aligned to the Amel_HAv3.1 reference genome^50^ using BWA-MEM^51^ v0.7.17. Reads were sorted with SAMtools^52^ v1.9 and duplicates marked (MarkDuplicates) with GATK^53^ v4.0.11.0. Variants for each sample were called using GATK’s HaplotypeCaller^54^ with the following non-default parameters–ERC GVCF,–sample-ploidy 1 and -A AlleleFraction. Joint variant calling was performed across all samples using GATK’s GenomicDBImport and GenotypeGVCFs with–sample-ploidy 1 and a window size of 2.5 Mb. The pipeline is implemented in BAGPIPE (https://bitbucket.org/renzo_tale/bagpipe/). Across the 1,407 drones we identified 21,203,582 variants. This included 15,847,428 SNPs, 6,316,757 INDELs, 1,139,661 other variant types (e.g. complex substitutions), and 5,126,225 multiallelic sites of which 2,324,214 were multiallelic SNPs. The mean genotype depth of coverage (DP) for these variants across sample-level averages was DP = 7.4 ± 7.5 (Fig. 1a). This reflects the various sequencing strategies employed by different studies from which the data was sourced, highlighting the need for careful filtering of variant, genotype and sample qualities.

### Variant quality validation and filtering

To ensure only high-quality variants were retained in AmelHap, variant calling metrics were evaluated and appropriate filters set. To this end, several key quality metrics were evaluated: Quality by Depth (QD), Root Mean Square Mapping Quality (MQ), Strand Odds Ratio (SOR), and genotype depth of coverage and quality (Fig. 1b–f).

QD is the variant confidence (QUAL) divided by the unfiltered depth of homozygous non-reference samples and is a more robust metric for filtering than either QUAL or depth (DP) alone. QD values generally fall within the range 0 to 40, however, where DP < 1 values of QD > 40 can be observed, thus, variants exceeding this threshold were filtered representing low-depth low-quality variants. We also set a lower end threshold for this metric, i.e. QD > 20 in order to remove spurious heterozygous variants^11^. This is because two peaks are typically observed from a diploid QD distribution (see GATK technical documentation on hard-filtering germline short variants), reflecting variants that are mostly observed in either the heterozygous (QD peak between 12–15) or homozygous state (QD peak between 28–32, in our case homozygous peak at QD = 29.5; Fig. 1b).

MQ is the root mean square mapping quality over all the reads at the site and is used to evaluate deviations in mapping quality. An MQ of 60 indicates good mapping qualities at the site, and the general recommendation for hard-filtering is to exclude sites with MQ < 40 (as above, see GATK technical documentation on hard-filtering). Within our data we observe a median MQ of 60, with 411,360 records (1.9%) having MQ < 40 and 882,951 (4.2%) having MQ < 50. Given that fewer than 5% of variants had an MQ < 50 we chose to filter on this higher stringency, rather than the generic MQ < 40.

SOR is a means of measuring strand bias, whereby one DNA strand is favoured over the other during sequencing, potentially resulting in a bias in evidence for one allele over the other. Intuitively strand bias should not present a problem with haploid data, however there is the potential for variants to fall within regions containing duplications which might then be influenced by such a bias. Removing the long tail of the SOR distribution seeks to remove sites demonstrating such bias. By filtering on SOR > 3 we remove 356,473 records (1.7%) from our data (Fig. 1c).

Overall, after evaluating the distributions of these quality metrics, and applying appropriate filtering (filter 1: bcftools filter -sLowQual -e’QD < 20 || QD > 40 || MQ < 50 || SOR > 3′), there remained 18,154,924 records, comprising 13,777,414 SNPs and 5,159,405 INDELs, including 2,974,414 multiallelic sites of which 298,587 were multiallelic SNPs.

### Sample quality validation and filtering

**Fig. 2:**
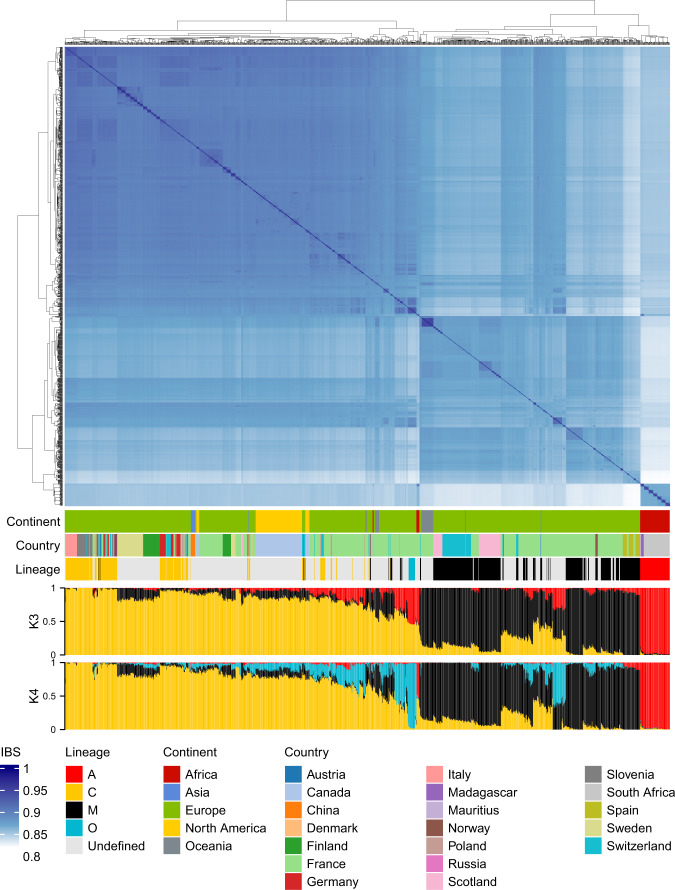
Identity by state (IBS) clustering and admixture estimates validate reported sample origins and relationships. Based on a dataset of 660 K variants after filtering on minor allele frequency (MAF) >1% and linkage disequilibrium (LD) r^2^ < 0.1 for variants up to 10 kb apart. We observe a median IBS of 0.87 ± 0.02 across all drones. Hierarchical clustering indicates three dominant clusters, corresponding to samples with reported ancestry from the A, C and M lineages. Dark blue squares adjoining the diagonal highlight closely related or sibling drones. Admixture levels at *K* = 3 and *K* = 4 were estimated with ALStructure. At *K* = 3, the *A. m. caucasia* samples have mixed ancestry estimates of C (~56%) and A (~44%) lineages, while at *K* = 4 this subspecies emerges as a distinct genetic background. Some samples originate from overseas territories of named countries, for instance New Caledonia is a French territory in Oceania, refer to sample metadata^57^ for detailed sampling locations. The lineage track refers to the sample lineage as reported in the study from which the sample originates.

Following variant quality validation, we evaluated sample-level genotype quality (GQ) across the 25,543,978,068 genotypes (Fig. 1e). GQ represents the Phred-scaled confidence that the genotype assignment (GT) is correct, and is the difference between the Phred-scaled likelihoods (PLs) of the most likely and second most likely genotypes. As the PLs are normalized, the most likely genotype has a PL of 0, thus GQ equates to the second most likely PL and is capped at 99. A Phred quality score of 20 is equivalent to a 1% probability of error, while a score of 99 equates to a percent error probability less than 1 × 10^−09^. GQ values were extracted with vcftools^55^ v0.1.13 (–extract-FORMAT-info GQ) for all sites that passed the initial filter (–remove-filtered-all). To evaluate the relationship between GQ and depth of coverage for a genotype, we also extracted DP values (–extract-FORMAT-info DP). Although we observe genotype depths in excess of 1,000, the significant majority (99.9%) have DP ≤ 704, equivalent to half the sample size, and corresponds to the elbow at the upper end of the distribution (Fig. 1d). We also observe a majority (91.7%) of genotypes to have GQ = 99 (Fig. 2f), with a median of GQ = 51 ± 29.7 s.d. across genotypes with DP ≤ 704. The GT error probability at GQ = 50 is 0.001%. Following these analyses we elected to set genotypes with either DP > 704 or GQ < 40 to missing (filter 2: bcftools + setGT ${VCF}–t q -i‘FORMAT/DP > 704 | FORMAT/GQ < 40’ -n “.”), thus retaining genotypes with 99.99% accuracy and excluding those in high coverage regions which could potentially result from duplications. After masking these genotypes and removing variants with a non-reference allele frequency (AF) of zero (filter 3) there remained 18,152,805 variants across the 1,407 drones. Applying to these high-quality variants a minimum 90% threshold for sample and variant call rates (filter 4) returns 1,328 samples and 17,414,346 variants, which forms the AmelHap (v1.1.1) dataset.

### Genetic identity of samples and population structure

We analysed pairwise identity by state (IBS) and genetic admixture to validate if these corroborated with reported or expected relationships. Prior to performing these analyses, we first filtered to retain variants with a minor allele frequency (MAF) >1% (bcftools view -f.,PASS–min-af 0.001:minor), then removed variants in strong linkage disequilibrium (r^2^ > 0.1) using bcftools + prune by considering pairs of variants up to 10 kb apart (–max 0.1 –window 10 kb). This left 660,258 records comprising 513,322 SNPs and 152,810 INDELs, including 31,921 multiallelic sites of which 2,980 were multiallelic SNPs. Using the LD filtered dataset, we generated an IBS matrix with Plink^56^ v1.90p (–distance square gz ibs). From this, we observe clear clustering of samples corresponding to C, M, and A lineage ancestries, as reported within the respective studies from which the samples were sourced (Fig. 2). In addition, we observe clusters of very closely related samples or siblings (dark blue squares along the diagonal in Fig. 2), most of which correspond to drones sampled from the same colony.

**Fig. 3:**
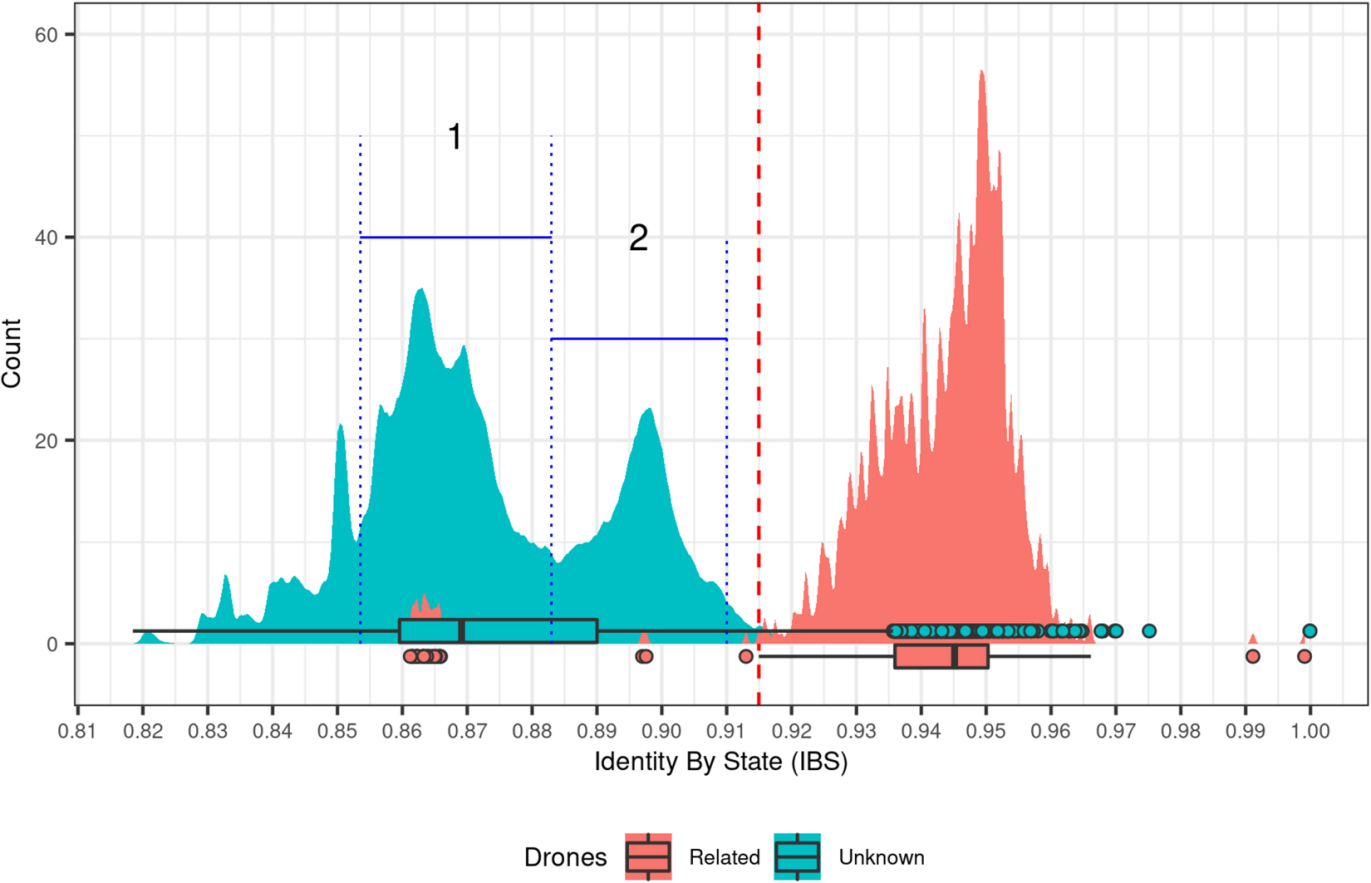
Identity by state (IBS) distributions delineate sample relatedness and evolutionary divergence between unrelated drone pairs. Based on a dataset of 660 K variants after filtering on linkage disequilibrium (LD). Drones were considered siblings if they were reported to have been sampled from the same colony. Dashed red line indicates lower whisker of drone sibling IBS values (IBS >0.914). A number of outliers of unknown relatedness fall within the sibling IBS range (blue circles). These outliers predominantly derive from colonies within a selection programme or were sampled from small, isolated populations which would explain their high genetic identity. Two dominant peaks within the distribution of samples of unknown relatedness are evident, which broadly correspond with evolutionary divergence: (1) the majority of drone pairs (54.9%) are between-lineage comparisons; (2) the majority of drone pairs (83.6%) are within-lineage comparisons.

To further investigate this point, we plotted the distribution of pairwise IBS values and colored them according to their reported relationship, *i.e*. pairs of drones from unrelated colonies or whose relatedness is unknown (blue), and pairs of drones reported to be siblings/sampled from the same colony (red) (Fig. 3). We find that both groups are clearly separated, with the majority of sibling drones having IBS > 0.914 (the lower whisker of their distribution), demonstrating that users can easily filter AmelHap based on IBS to reduce any bias in downstream analyses linked to family relatedness. A small number of sibling drone pairs (2.5%) fall below this threshold, however, these outliers are not unexpected, as it is possible for drones from the same queen to inherit complementary haplotype blocks, thus effectively only sharing genomic regions that are homozygous in the queen. Similarly, a number of drone pairs sampled from different colonies returned IBS values exceeding the drone sibling lower whisker. These outliers represent a very small proportion of unrelated drone pairs (0.66%). The higher genetic relatedness among these samples is not unexpected due to the composition of the dataset - which includes drones from several breeding programmes, single apiaries and isolated or experimental populations (Supplementary Table 1). For end-user convenience, we have labelled outlier drones which return more than 3 high IBS values (column ‘IBS Risk’ in metadata^57^).

Pairwise IBS values between the unrelated drones are roughly distributed across two dominant peaks (Fig. 3, blue). Considering only the 317 drones whose evolutionary lineage was reported, the majority of pairs (54.9%) under peak 1 involve comparisons between drones reported to be from different lineages, while drone pairs under peak 2 are dominated by within-lineage comparisons (83.6%). Although these figures are skewed to an extent by the limited sample representation for each lineage, they broadly demonstrate that the IBS peaks correlate with genetic divergence between and within lineages as reported in previous studies^7,9^.

We next evaluated the overall genetic structure of AmelHap by calculating global ancestry estimates (Q) with ALStructure^58^ (https://github.com/StoreyLab/alstructure) using default settings. ALStructure unifies PCA-based and likelihood-based methods for estimating ancestry by first clustering the data using latent subspace estimation (LSE), a method similar to PCA, and then estimating a sample’s global ancestry proportions using the alternating least squares (ALS) method to transform the allele frequencies obtained from LSE. We ran ALStructure on the LD filtered dataset for *K* = 2 to 6 dimensions and applied the structural Hardy-Weinberg Equilibrium (sHWE) test^59^ to generate p values from each *K* run using 3 null datasets. For the purpose of the sHWE test, missing genotypes were set to 0.5 as the function cannot process matrices with missing data. The resulting sHWE p values were applied to the entropy-based procedure described by Hao and Storey^59^, in order to determine the optimal *K*. The resulting entropy values for *K* = 2 to 6, respectively, were 763, 766, 768, 769, and 770, which, although indicating *K* = 2 to be optimal, demonstrates only marginal difference at these *K* levels. We present the results of *K* = 3 and *K* = 4 (Fig. 2) based on there being four reported lineages within the dataset with the caveat that one of these lineages (O) is poorly represented. Global ancestry estimates at *K* = 3 corroborate the reported sample lineages A, C and M (Fig. 2), with the few representatives of the O lineage (*A. m. caucasia*) having mixed ancestry estimates of C (~56%) and A (~44%) lineages. At *K* = 4 a distinct background emerges for these *A. m. caucasia* samples. We also observe a number of samples whose admixture-inferred ancestry is inconsistent with their reported lineage, highlighting the need to apply caution when processing third party data.

## Data Records

Sequence data generated in this study have been deposited under project accession code PRJEB39369^49^. ENA accession numbers to individual sequence data are deposited at Zenodo^57^, listing all 1407 drone sequences processed in this paper alongside sample metadata (i.e. sample origin, siblings, reported type/subspecies) and variant filtering metrics (i.e. genotype depth and missingness). Raw gVCF files for all samples grouped by project accession^60–67^, along with joint-called raw variants^68–74^ across samples from these project accessions are available from the AmelHap community on Zenodo (https://zenodo.org/communities/amelhap; Supplementary Table 2). The AmelHap_1.1.1.f4^75^ VCF file follows filter 4, and includes common variant INFO (AC, AF, QD, etc.) and individual FORMAT (GT:AD:DP:GQ) annotations allowing users to further filter the dataset according to their needs. This VCF file has also been deposited at the European Variation Archive^76^ (Project Accession PRJEB59912^77^). Sample metadata is also available from the AmelHap community^78^, where revisions will be uploaded as the resource grows. The genetic maps used for imputation and example code to process sequence data, filter variants, and generate plots is available at https://bitbucket.org/gibberwocky/amelhap.

## Technical Validation

### Genotype imputation

One of the key values of a HapMap is its utility for imputing missing genotypes. To evaluate AmelHap in this regard, we tested its imputation performance on a subset of drones and an independent diploid dataset.

**Fig. 4:**
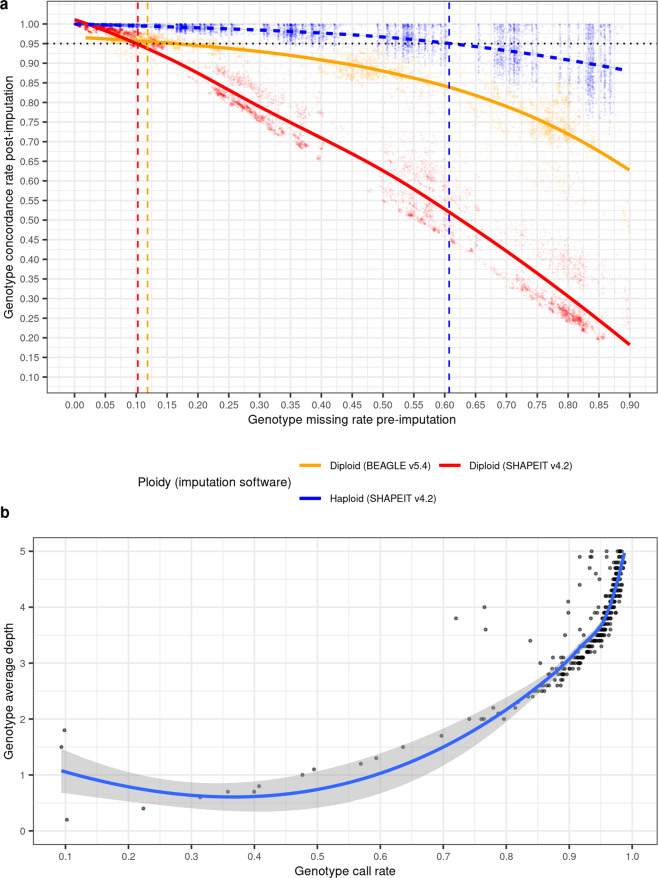
Plotting genotype missing versus concordance rate demonstrates the value of AmelHap for genotype imputation. (**a**) Data are presented for three imputation datasets: (1) haploids (blue) and (2) diploids (orange) imputed using SHAPEIT (orange), and (3) diploids imputed using Beagle (red). Haploid results are based on imputing 100 unrelated drones within AmelHap. For each of the 100 drones, *N* variants were randomly pruned in 1 Mb windows, with *N* ranging from 12.5 K to 750 K SNPs. Following self-imputation with the AmelHap data, genotypes were compared against the truth dataset. Diploid results are based on imputing 139 unrelated workers. For each worker *N* variants were randomly pruned in 1 Mb windows, with *N* ranging from 10 K to 100 K SNPs. Following self-imputation with AmelHap, imputed genotypes were compared against the original genotypes. The dotted horizontal line indicates a 95% concordance threshold. Each point represents concordance data for a single sample and chromosome from one of the pruned genotype sets. A localised regression (loess) trend line is presented across each imputed dataset, with intercepts at *y* = 0.95 of *x* = 0.61 for haploids, *x* = 0.10 for diploids imputed with SHAPEIT and x = 0.12 for diploids imputed with Beagle. (B) Genotype average depth versus call rate (see metadata^57^ filter 3 variants) indicates that sequencing to >3X genotype depth of coverage typically results in >90% call rate. For clarity the y axis has been limited to a maximum depth of 5, resulting in data for 1063 samples being excluded from the plot - of which 56 had a call rate <90%.

For the imputation of a subset of haploid drones, we first subset and filtered AmelHap to retain bi-allelic SNPs which left more than 11 M SNPs positioned along the 16 linkage groups. Imputation analyses were conducted with SHAPEIT^79^ v4.2 (–sequencing) using genetic maps generated by Wragg *et al*.^8^. We generated a “truth” dataset by self-imputing missing genotypes in AmelHap. Then, we randomly selected for imputation 100 unrelated samples (see metadata^57^). Each sample was extracted from AmelHap and processed independently with bcftools + prune to randomly remove *N* SNPs in 1 Mb windows (-w 1000000 bp -n ${N} -N rand). We repeated this for *N* = 12.5 K, 25 K, 37.5 K, 50 K, 62.5 K, 75 K, 87.5 K, 100 K, 250 K, 500 K, and 750 K SNPs. For each *N*, the pruned samples replaced their equivalent selves in a copy of the AmelHap truth dataset and self-imputation was performed. We then calculated genotype concordance by comparing the imputed versus truth genotypes with bcftools stats. Figure 4a presents the relationship between pre-imputation missingness and post-imputation genotype concordance, indicating that >95% genotype concordance is achieved when imputing samples with up to 61% missing genotypes in 1 Mb windows, demonstrating the utility of AmelHap for high-accuracy imputation in drones.

We next sought to evaluate its effectiveness for genotype imputation in diploids. A dataset of 139 diploid worker honey bees^7^ comprising samples from 13 subspecies representing 4 evolutionary lineages (A, C, M and O) was sourced from the CNGB Sequence Archive (CNSA) of the China National GeneBank DataBase (CNGBdb accession number CNP0001986^80^). The sequence data was processed to generate gVCF files^81,82^ as per the drone data with a single exception - variant calling was performed with–sample-ploidy 2. Joint calling of this dataset included AmelHap as the target intervals (GATK GenotypeGVCFs–intervals). Each sample within the diploid dataset was processed independently with bcftools + prune to randomly remove *N* SNPs in 1 Mb windows (-w 1000000 bp -n ${N} -N rand). We repeated this for *N* = 10 K to 100 K SNPs, in 10 K increments. For each *N*, the pruned samples were merged with the AmelHap truth dataset and self-imputation was performed. We then calculated genotype concordance in the diploids by comparing their imputed versus original genotypes with bcftools stats. While not as effective at imputation in the diploid dataset, >95% genotype concordance is achieved when up to 10% of data is missing (Fig. 4a). To establish if the reduced performance was a consequence of the data structure (i.e. homozygotised haploid genotypes) or the software, we repeated imputation on the same pruned data with Beagle^83^ v5.4, using the pruned data as the target panel and AmelHap as the reference panel, and evaluated the results in the same manner. Imputation with Beagle returned >95% genotype concordance when up to 12% of data was missing and performed substantially better than SHAPEIT at higher levels of missingness (Fig. 4a). Thus, while still being less effective than for haploids, diploid samples with a call rate of >90% achieve 95% genotype concordance. When considering genotype average depth versus call rate (see metadata^57^ filter 3 variants), a call rate of >90% is typically achieved in samples with >3X genotype depth of coverage (Fig. 4b), highlighting the suitability of AmelHap for low-pass whole-genome sequencing of diploids.

Finally, of note, is that imputation performance differed according to the subspecies tested, with concordance ranging on average across the different missingness runs from 0.88 to 0.93. The subspecies returning the lowest concordance was *A. m. ruttneri*, for which no drones were available for inclusion in AmelHap. The highest imputation performance was observed in *A. m. mellifera* which is well represented within AmelHap.

## Usage Notes

We have demonstrated that by using AmelHap to impute high levels of missing data (61%), very high genotype concordance ( > 95%) can be achieved in drones. We also demonstrate the resource to be effective at imputing moderate levels of missing data (12%) in an independent diploid dataset. We have not extensively investigated the parameter space for imputation, or the full range of tools available, and so further improvements on imputation performance are likely achievable. Moreover, restricting the reference panel to lineages relevant to the target panel may further increase accuracy. Based on the AmelHap filter 3 metrics (see metadata^57^), sequencing to at least 3X coverage should achieve a 90% call rate (Fig. 4b), demonstrating that AmelHap will be a useful resource for genotype imputation in low pass whole-genome sequencing.

Beyond supporting phasing and genotype imputation, AmelHap can serve as a valuable reference to support studies of population genetics, including hypothesis testing on population differentiation and the evolutionary history of the species. It will also be an important resource for assay development in relation to the identification of ancestry-specific markers.

There remains an ongoing need to improve on the limited genomic resources for the species. As has been demonstrated in other species, increasing the sample size and diversity of AmelHap will yield improvements in genotype imputation^84,85^. As such, we aim to continue to develop the resource and have provided details on a Zenodo community for AmelHap (https://zenodo.org/communities/amelhap/about/) to enable researchers to easily replicate the sample processing steps, to generate a haploid gVCF file from raw fastq data, using the Nextflow sarek workflow (https://nf-co.re/sarek). We aim to integrate gVCF files uploaded to the AmelHap community on a regular basis, enabling the community to grow the resource for the benefit of all. Looking ahead, the data underpinning AmelHap can be leveraged further to support the development of a pan-genome^86^. A graph genome incorporating high quality assemblies from each of the evolutionary lineages, integrating haplotypes representative of variation within those lineages, would enable a greater range of genetic diversity to be captured. In particular, it would allow structural variations to be genotyped which, to date, remain largely understudied in the species.

## Supplementary information

Supplementary Information

## Data Availability

Code underpinning the pipeline (BAGPIPE) used for aligning sequence data and calling variants BAGPIPE is available at https://bitbucket.org/renzo_tale/bagpipe/. Detailed code outlining the various analyses and parameters is available without restriction at https://bitbucket.org/gibberwocky/amelhap. All analyses have been performed with freely available software. These include: BWA-MEM v0.7.17; SAMtools v1.9; GATK v4.0.11.0; vcftools v0.1.13; bcftools v1.13; Plink v1.90p; R^87^ v4.1.3; R packages (vcfR^88^, alstructure, ggplot2, ggpubr, ggdist, tidyverse, readxl, reshape2, ComplexHeatmap, circlize, paletteer); and SHAPEIT v4.2. With the exception of plotting data, all analyses were conducted on the Edinburgh Compute and Data Facility (ECDF), a high performance computing cluster (HPC) running a Linux operating system.
