# Supplementary material for: AmelHap: Leveraging drone whole-genome sequence data to create a honey bee HapMap
Source: Sci Data. 2023 Apr 10;10:198. doi: 10.1038/s41597-023-02097-z (PMC10086014; doi:10.1038/s41597-023-02097-z)
Supplement: Supplementary file 1 — Supplementary Information [file 41597_2023_2097_MOESM1_ESM.pdf]

Supplementary Table 1. Overview of sample origin, sampling, and sequencing design and strategy of the collated drone sequence data included in AmelHap.

Study	Project Accession	Country of origin	Reported type	# Samples	# Colonies	Sampling scheme	Illumina sequencing platform	Library
Harpur et al. 2019	PRJNA363032 [41]	**Canada**	selected (hybrid)	125	41	Average of 3 drones per colony	HiSeq 2500	2 x 150 bp
		**Germany**	*carnica*	18	18	Single colonies		
			caucasia	15	15	Single colonies from a French apiary, imported from Georgia		
		**France**	hybrid	202	202	Single colonies from various breeders throughout France		
			selected (hybrid)	65	65	Single colonies from royal jelly breeders		
			mellifera	142	112	Single colonies sampled from *A. m. mellifera* conservatories		
		**France**	hybrid	158	40	Average of 4 drones per colony (MOSAR experiment)		
		**Corsica (France)**	hybrid	44	44			
		**New Caledonia (France)**	hybrid	40	40			
		**Italy**	*ligustica*	30	30			
Wragg et al. 2016; Wragg et al. 2017; Wragg et al. 2022	PRJNA311274 [38]	**Poland**	*carnica*	19	19		HiSeq 2000, 2500 and 3000	TruSeq Nano (2 x 100 bp or 2 x 125 bp)
		**Denmark**	unreported	2	2			
		**China**	hybrid	10	10			
		**Madagascar**	*unicolor*	6	6	Single colonies		
		**Mauritius**	*unicolor*	2	2			
		**Rodrigues (Mauritius)**	hybrid	2	2			
		**Reunion (France)**	hybrid	6	6			
		**Scotland**	*mellifera*	28	28			
		**Spain**	*iberiensis*	30	30			
		**Slovenia**	*carnica*	20	20			
		**Austria**	*carnica*	5	5			
		**Austria**	*mellifera*	2	2			
		**Germany**	*carnica*	33	33			
Jones et al. 2020	PRJNA596071 [40]	**Germany**	*mellifera*	3	3	Single colonies		
		**Slovenia**	*carnica*	12	12			
		**Norway**	*mellifera*	4	4			
		**Switzerland**	*mellifera*	2	2			
-	PRJNA578233 [47]	**Russia**	*mellifera*	3	3	Single colonies	HiSeq 2500	
This study	PRJEB39369 [49]	**Scotland**	hybrid	45	9	5 Drones per colony from 5 apiaries in Midlothian	NovaSeq	TruSeq Nano (2 x 150 bp)
			Buckfast	16	16			
Parejo et al. 2016	PRJEB16533 [46]	**Switzerland**	*carnica*	31	31	Single colonies	HiSeq 2500	TruSeq Nano (2 x 125 bp)
			mellifera	72	72			
		**Sweden**	hybrid	78	8	9-10 Drones sampled from 8 colonies on Gotland	HiSeq 2500	TruSeq PCR-free (2 x 125 bp)
		**Finland**	hybrid	80	8	10 Drones from 8 colonies from Åland islands		
Kawakami et al. 2019	PRJNA516678 [43]	**South Africa**	*capensis*	28	3	9-10 Drones sampled from 3 colonies per subspecies	HiSeq 2500	TruSeq Nano (2 x 125 bp)
			scutellata	29	3			
**Total**				**1407**	**946**			

Supplementary Table 2. Raw and filtered data generated, including filter description, statistics and DOIs. Filter 1 identified low-quality variants including retaining those whose quality by depth (QD) indicated that sequencing reads strongly supported a single allele at the variant. Filter 2 set genotypes as missing if there was more than 0.01% chance of error, or if their sequencing depth was greater than 99.9% of other genotypes - potentially indicating the variant to fall within a duplication. Filter 3 removed variants identified as low quality by filter 1, in addition to any variants that had a minor allele frequency of 0 after the masking applied by filter 2. Filter 4 removed samples and variants with call rate <90%, while a final filter removed variants in strong linkage disequilibrium (LD, pairwise r2 ≤ 0.1 at distances up to 10 kb). Note that if a record contains both a SNP and an INDEL then it contributes to both counts in bcftools stats, hence the numbers presented do not sum to the total number of variants identified.

Data	Filters applied	Samples	Variants	SNPs	INDELs	Other	Multiallelic variants	Multiallelic SNPs	ts/tv	Zenodo DOI	ENA Project Accession
Raw	None	1407	21203582	15847428	6316757	1139661	5126225	2324214	4.36	10.5281/zenodo.6669803 [68]<br>10.5281/zenodo.6669889 [69]<br>10.5281/zenodo.6669899 [70]<br>10.5281/zenodo.6669907 [71]<br>10.5281/zenodo.6669919 [72]<br>10.5281/zenodo.6669929 [73]<br>10.5281/zenodo.6669952 [74]	PRJEB16533 [46]<br>PRJEB39369 [49]<br>PRJNA311274 [38]<br>PRJNA363032 [41]<br>PRJNA516678 [43]<br>PRJNA578233 [47]<br>PRJNA596071 [40]
Filter 1	Excludes variants with quality by depth (QD) less than 20 or greater than 40, or with mapping quality (MQ) less than 50, or with a strand odds ratio (SOR) greater than 3.	1407	18154924	13777414	5159405	-	2974414	298587	4.74	-	-
Filter 2	Genotypes with depth (DP) greater than 704 or quality (GQ) less than 40 as set to missing.	1407	18154924	13777414	5159405	-	2974414	298587	4.74	-	-
Filter 3	Monomorphic variants removed.	1407	18152805	13775419	5159281	-	2974413	298587	4.74	-	-
Filter 4 (AmelHap_1.1.1.f4)	Excludes samples and variants with a call rate less than 90%.	1328	17414346	13272369	4820365	-	2575237	284755	4.85	10.5281/zenodo.7615208 [75]	PRJEB59912 [77]
